# Relative importance of long‐term changes in climate and land‐use on the phenology and abundance of legume crop specialist and generalist aphids

**DOI:** 10.1111/1744-7917.12585

**Published:** 2018-05-17

**Authors:** Martin Luquet, Maurice Hullé, Jean‐Christophe Simon, Nicolas Parisey, Christelle Buchard, Bruno Jaloux

**Affiliations:** ^1^ Agrocampus Ouest, Centre of Angers, Institute of Genetics Environment and Plant Protection (IGEPP—Joint Research Unit 1349) Angers France; ^2^ INRA, Institute of Genetics Environment and Plant Protection (IGEPP—Joint Research Unit 1349) Le Rheu France

**Keywords:** abundance, climate warming, degree of specialization, land‐use change, legume aphids, phenology

## Abstract

Insect populations are prone to respond to global changes through shifts in phenology, distribution and abundance. However, global changes cover several factors such as climate and land‐use, the relative importance of these being largely unknown. Here, we aim at disentangling the effects of climate, land‐use, and geographical drivers on aphid abundance and phenology in France, at a regional scale and over the last 40 years. We used aerial data obtained from suction traps between 1978 and 2015 on five aphid species varying in their degree of specialization to legumes, along with climate, legume crop area and geographical data. Effects of environmental and geographical variables on aphid annual abundance and spring migration dates were analyzed using generalized linear mixed models. We found that within the last four decades, aphids have advanced their spring migration by a month, mostly due to the increase in temperature early in the year, and their abundance decreased by half on average, presumably in response to a combination of factors. The influence of legume crop area decreased with the degree of specialization of the aphid species to such crops. The effect of geographical variation was high even when controlling for environmental variables, suggesting that many other spatially structured processes act on aphid population characteristics. Multifactorial analyses helped to partition the effects of different global change drivers. Climate and land‐use changes have strong effects on aphid populations, with important implications for future agriculture. Additionally, trait‐based response variation could have major consequences at the community scale.

## Introduction

In a context of global changes, the distribution, abundance and phenology of insect populations are predicted to be profoundly modified, notably over regional scales, through the permanent alteration of environmental factors such as climate and landscape (Moraal & Jagers Op Akkerhuis, 2011; Eskildsen *et al*., 2013; Aguirre‐Guttiérez *et al*., 2016). Pest insect populations are expected to show major responses, as they are exposed to both climate change and large alterations of their habitat through globalization, which modifies crop distribution and agricultural practices from local to worldwide scales (Moraal & Jagers Op Akkerhuis, 2011; Wang *et al*., 2015). Climate change (e.g., temperature increase, alteration of extreme events, and precipitation patterns) is known to affect pest insects in many direct and indirect ways, including alteration of phenology and population dynamics (Jamieson *et al*., 2012), and shifts in geographical distribution (Bebber, 2015). In temperate regions, reduced development time and strengthened overwintering ability are notably recorded (Bale *et al*., 2002), leading to increased population growth rates and number of generations produced every year (Powell & Bentz, 2009; Altermatt, 2010). In addition, globalization can lead to a sizeable increase in the proportion and area of a given crop in a region, together with higher pest populations (Tilman, 1999; Ouyang *et al*., 2014; Rand *et al*., 2014), and increased insect dispersion (Tscharntke *et al*., 2005; Wallner *et al*., 2014). Previous studies have shown variable impacts of climate and land‐use changes on insect populations at different regional scales (Fox *et al*., 2014; Aguirre‐Guttiérez *et al*., 2016), highlighting the difficulty of disentangling their respective influences. Indeed, land‐use and climate change effects frequently overlay (Fox *et al*., 2014), or interact with each other (Oliver & Morecroft, 2014), and it may be challenging to successfully attribute insect responses to the corresponding factors (Kiritani, 2007). It is thus important to consider both these factors to understand variation in insect population characteristics in a spatiotemporal context, using appropriate tools and datasets to partition their contributions and their interactions (Juroszek & von Tiedemann, 2013).

In temperate regions, aphids are major pests, whose population characteristics are prone to be affected by long‐term regional changes (Hullé *et al*., 2010). Many aphid species migrate from winter refuges to crops in spring through formation of winged morphs, with a high reliance of migration dates on winter temperature (Bale *et al*., 1988, 2002). Previous studies have established a tight relationship between winter temperature increase and aphid advancements in dates of first flight, leading to earlier arrivals on crops (Hullé *et al*., 1994; Harrington *et al*., 2007; Bell *et al*., 2015). Though results are less significant, climate change can affect annual aphid abundance as well (Bell *et al*., 2015). Aphid numbers may also be influenced by land‐use: indeed, it seems that geographical patterns of aphid abundance are related to regional landscape features, including the proportion of a given crop in a given area (Cocu *et al*., 2005a, b). However, besides theoretical models (Ciss *et al*., 2014), little is known about the relationship between aphid population characteristics and the area of crop, notably at a large scale, and land‐use data are needed to improve predictions of global change impacts (Ryalls & Harrington, 2017). Because so few studies have considered both parameters at once (Cocu *et al*., 2005b; Wang *et al*., 2015), the respective influence of climatic and land‐use factors on aphid spatiotemporal population dynamics is poorly documented. Besides, their effects probably differ among species: Harrington *et al*. (2007) suggested that response variation observed in aphid species advancement in first flight dates could be trait‐based, linked to the insects’ biology. A recent study from Bell *et al*. (2015) seems to indicate that aphid trait variation, such as reproduction mode or life cycle, could have an impact on aphid responses to warming. In addition to climate change, modifications of cultivated areas are expected to induce differential responses among species (Andersson *et al*., 2013). Traits such as dispersal capacity or habitat specialization could be major drivers of insect responses to resource density variation (Öckinger *et al*., 2010).

In this study, we explored the respective influence of climate, land‐use, and geographical context on aphid abundance and phenology, over a large scale (France) and in a context of long‐term changes. To tackle this issue, we used standardized, long‐term and spatially explicit datasets on flying aphids from different species caught in suction traps from 1978 to 2015. Such datasets are particularly well suited to discriminate the impacts of different global change drivers (Juroszek & von Tiedemann, 2013) and to consider geographical variation (Cocu *et al*., 2005b). General and specific aphid responses to environmental changes were evaluated. The degree of plant specialization of the aphids was studied as an additional factor potentially affecting the response to global changes.

We focused our work on aphid species showing different levels of host‐plant preferences for protein‐rich crops. Protein‐rich crops are Fabaceae (legumes) plants that are mainly grown for feeding livestock. In France, the main cultivated protein crops are pea (*Pisum sativum*), faba bean (*Vicia faba* var. *minor*), and lupin (*Lupinus* spp*.)*. Although their production used to be quite important, growing dependence on American soy importation, associated with diseases and bad climatic conditions, led French production to collapse at the turn of the century (Häusling, 2011; Bues *et al*., 2013). Temporal change in protein crop production, associated with its geographical variation (most of the fields are grown in the North and West of the country) and climate change make these crops good models to study global change effects on aphid spatiotemporal patterns. In addition, European Union policies are currently aiming at boosting protein crop farming (Bues *et al*., 2013; de Visser *et al*., 2014; Murphy‐Borken *et al*., 2015; Bourget, 2017). These policies, combined with climate change, are likely to induce responses from legume pests, which could be different from those experienced in the past. Earlier migration dates can make plants more sensitive to aphid‐borne viruses (Canto *et al*., 2009; Ryalls & Harrington, 2017) and exposed to a longer risk period, and increased aphid abundance can lead to greater plant damage (Macedo *et al*., 2003). From an agricultural standpoint, understanding the main trends that drive pest populations is also helpful to predict future pest pressure, giving insight to set up adapted control methods for securing protein crop production.

## Materials and methods

### The Agraphid suction trap network

**Figure 1:**
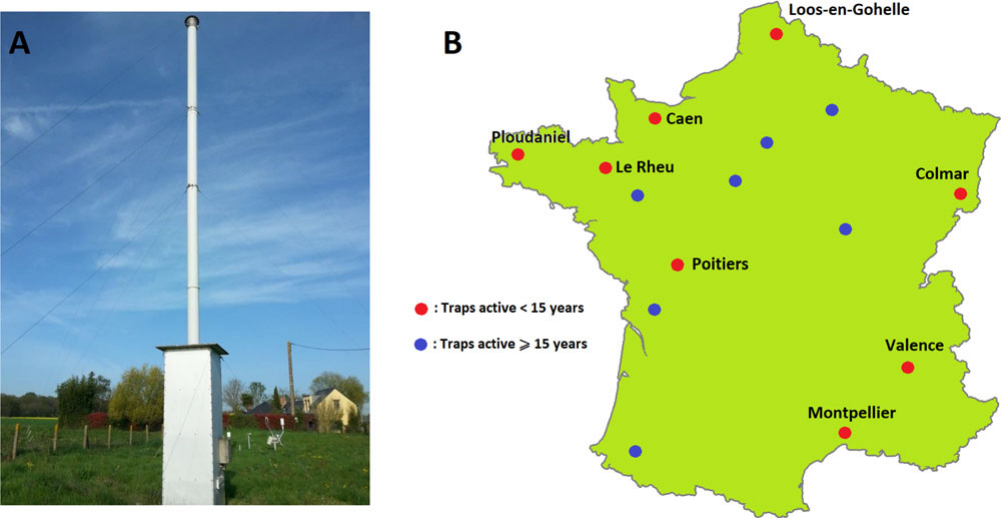
The Agraphid suction trap network. (A) Trap located at Domaine de la Motte INRA, Le Rheu, France (Picture by Melissa Anne). (B) Trap locations.

Agraphid is a French network of suction traps integrated into the European EXAMINE network (Harrington *et al*., 2004). These standardized traps (Fig. 1A) capture arthropods at 12.2 m above the ground, by vacuuming the air (Macaulay *et al*., 1988), and are representative of the aerial population over a wide area (Taylor & Palmer, 1972; Hullé & Gamon, 1989; Cocu *et al*., 2005a). Since the installation of the first trap in 1978, the Agraphid network has facilitated the collection of daily data on more than 200 aphid species in 15 locations all over France, although the number of active traps varied over time (Hullé *et al*., 2011; Fig. 1B).

### Biological data (aphids)

All aphid data used came from the eight Agraphid traps that have been active for at least 15 consecutive years (Fig. 1B), in order to have the most balanced possible dataset and to avoid bias due to too small samples. Five aphid species were studied, which are all protein crop pests with different degrees of specialization to their hosts (Blackman & Eastop, 2000; Hullé *et al*., 2006): *Acyrthosiphon pisum* (specialist of Fabaceae), *Aphis craccivora*, *Aphis fabae* (both generalists with preference for Fabaceae), *Macrosiphum euphorbiae* (generalist, ≈ 20 plant families) and *Myzus persicae* (generalist, >50 plant families). *A. pisum* and *A. fabae* can however show intraspecific host range variation as they are complexes of different biotypes or subspecies (Peccoud *et al*., 2015; Vorburger *et al*., 2017). Studied species were chosen for being sufficiently abundant: they were present in all traps and had a mean annual abundance >20 in at least six traps. Other aphid protein crop pests, such as the lupin aphid *Macrosiphum albifrons*, were not selected because they did not fulfill these requirements. Biological data obtained (years and traps used) are summarized in Table 1.

**Table 1:** Summary of data used. *T*_min_: minimum daily temperature, *T*_max_: maximum daily temperature, Rain: daily rainfall, Wind: average daily wind speed

	Activity of traps								Crop area			
Year	Loos	Caen	Colmar	Ploudaniel	Le Rheu	Montpellier	Poitiers	Valence	Pea	Faba bean	Lupin	Climate (*T*_min_, *T*_max_, Rain, Wind)
1978	X		X	X	X	X		X				X
1979	X		X	X	X	X		X				X
1980	X		X	X	X	X		X				X
1981	X		X	X	X	X		X				X
1982	X		X	X	X	X		X				X
1983	X		X	X	X	X		X				X
1984	X		X	X	X	X		X				X
1985	X		X	X	X	X		X				X
1986	X	X	X	X	X	X		X				X
1987	X	X	X	X	X	X		X				X
1988	X	X	X	X	X	X		X				X
1989	X	X	X	X	X	X	X	X	X	X	X	X
1990	X	X	X	X	X	X	X	X	X	X	X	X
1991	X	X	X	X	X	X	X	X	X	X	X	X
1992	X	X	X	X	X	X	X	X	X	X	X	X
1993	X	X	X	X	X	X	X	X	X	X	X	X
1994	X	X	X	X	X	X	X	X	X	X	X	X
1995	X	X	X	X	X	X	X	X	X	X	X	X
1996	X	X	X	X	X	X	X	X	X	X	X	X
1997	X	X	X		X	X	X	X	X	X	X	X
1998	X	X	X		X	X	X	X	X	X	X	X
1999	X	X	X		X	X	X	X	X	X	X	X
2000	X	X	X		X	X	X	X	X	X	X	X
2001	X		X		X	X	X	X	X	X	X	X
2002	X		X		X		X	X	X	X	X	X
2003	X		X		X		X	X	X	X	X	X
2004	X				X		X	X	X	X	X	X
2005	X				X		X	X	X	X	X	X
2006	X				X		X	X	X	X	X	X
2007	X				X		X		X	X	X	X
2008					X		X		X	X	X	X
2009					X		X		X	X	X	X
2010					X		X		X	X	X	X
2011					X				X	X	X	X
2012					X				X	X	X	X
2013					X				X	X	X	X
2014					X				X	X	X	X
2015					X				X	X	X	X

Sources: Capture data: Agraphid, Crop area: SSP (Agreste), and Climatic data: CLIMATIK (INRA).

For each species, we derived insect abundance from weeks 15 to 32 per trap per year, which encompass the growing season of protein crops in all French regions (Terres Inovia, 2017). This period also covers almost all aphid captures during spring and summer flights (>98%). We chose date of 5th capture (D5C) as a phenological indicator of spring initiation, rather than first capture (Cocu *et al*., 2005b; Harrington *et al*., 2007), as some aphid annual first catches corresponded to isolated captures during winter. The use of D5C allowed reduction of noise while still being highly correlated with the date of 1st capture. Because the probability of capture depends on the abundance of insects in the near environment of the trap, particularly when few insects are present, D5C data were not taken into account when the abundance during the growing season was <10 individuals. This threshold was chosen after looking at the data graphically, as no spring flight peak could be clearly defined with less than 10 aphids trapped during this period. Phenological data were neither retained when, because of a mechanical failure, a particular trap could not operate in the 10 d before the date of 5th capture. As a result, the number of species‐trap‐years data available was 967 for the study of abundance and 816 for D5C. Data for 151 885 aphid individuals were analyzed.

### Environmental data

#### Land‐use data

Data corresponding to the cultivated area of proteaginous pea, faba bean, and lupin crops were gathered from the French Annual Agricultural Statistics, which synthesizes various governmental surveys (Agreste platform, http://agreste.agriculture.gouv.fr/). We collected all the data we could find, corresponding to annual cultivated surfaces (ha) of each crop between 1989 and 2015, in all regions (French “departments”) containing a suction trap (Table 1). This spatial scale (5993 ± 1122 km²) virtually corresponds to an 85 km‐diameter circle, which has been estimated to match the trap area of representation (Hullé & Gamon, 1989). No available, standardized dataset could be found before 1989 at such a scale.

#### Climatic data

Daily climatic data came from the closest INRA meteorological stations to the traps, that is, <25 km in most cases (CLIMATIK platform, https://www6.paca.inra.fr/agroclim/Les-outils). Four variables were extracted: minimum daily temperature (*T*_min_), maximum daily temperature (*T*_max_), daily rainfall (Rain), and wind speed (Wind). An estimated mean temperature (*T*_mean_) was calculated by computing (*T*_min_ + *T*_max_)/2. Climatic data were available for all periods of trap activity (Table 1), except for some wind speed data that were missing in the early 1980s.

### Data analysis

#### Preselection of climatic variables

A preselection of relevant variables was performed using the software tool CritiCor (Pierre *et al*., 1986), based on Goldwin's correlograms (Goldwin, 1982), to find critical periods during which a biological variable is tightly correlated with meteorological factors. We determined the correlations between biological variables (abundance, D5C) and the sum or average of weather variables (*T*_mean_, Rain, Wind) on all periods of the year, with all possible start dates and durations. We also considered cumulative temperature above thresholds varying between 12 and 16 °C, corresponding to most aphids’ lower flight threshold (Irwin *et al*., 2007). For each relationship 5000 Monte–Carlo simulations were run to assess correlation strength. Selected variables are presented in Table 2A. As Rain and Wind variables showed no clear relationship with any aphid species, they were removed from further analysis.

**Table 2:** Preselected variables for analyses. (A) Climatic variables identified as being potentially influencial on aphid date of 5th capture (D5C) or abundance during critical periods (CRITICOR; Pierre *et al*., 1986). Number of frost days is calculated as the number of days on which the daily mean temperature (*T*_mean_) dropped below 0 °C. (B). Fixed effect terms included in the models after a variance inflation factor variable dropping procedure (Zuur *et al*., 2010)

	Explained variable	
	D5C	Abundance
**A**. Potential determining climatic variables	**TJFMA**: Sum of degree‐days above 0 °C from January to April **TMA16**: Sum of degree‐days above 16 °C in March and April **FrJFM**: Number of frost days from January to March	**TJFMA**: Sum of degree‐days above 0 °C from January to April **FrJFM**: Number of frost days from January to March **TMAY**: Temperature in May
**B**. Fixed variables included in the models	**Pea** (pea regional area) **Faba bean** (faba bean regional area) **TJFMA** **TMA16** **Latitude** **Longitude**	**Pea** (pea regional area) **Faba bean** (faba bean regional area) **TJFMA** **TMAY** **Latitude** **Longitude**

#### Changes in biological and environmental parameters over time

Smoothing splines were used to visualize the average evolution of aphid abundance and phenology over time, as well as environmental variables. To explore the dynamics of annual aphid phenology, linear mixed models (LMM) were fitted, with D5C as the response variable, year as an explanatory variable, and trap as a random term. One model was fitted to all species data to get an average linear rate of change (with an additional “species” random term), along with one model per species. A LMM was also fitted to quantify average winter temperature evolution (TJFMA, Table 2A).

#### Effects of environmental drivers on aphid abundance and phenology

Relationships between aphid captures and agricultural, climatic, and geographical variables were explored using LMM and generalized linear mixed models (GLMM), with the lme4 package (Bates *et al*., 2015). For both biological variables, one general model containing all aphid data was fitted, along with one model per species to analyze their different responses. In each case, a full model was first built with D5C or abundance as the response variable. Climatic and land‐use factors were included as fixed explanatory variables, as well as trap latitude and longitude. We dropped highly correlated predictors to avoid multicollinearity, using a dropping procedure based on the variance inflation factor as suggested by Zuur *et al*. (2010) (VIF threshold = 5). Variables finally considered are listed in Table 2B. Two‐way interactions between land‐use and climatic variables were also included. The random part of the models consisted of a “Trap” term to account for correlation due to repeated measurements, a “Time” term to account for temporality, and a “Species” term for the “all‐species” models. From every full model, we used an all‐possible‐regressions selection procedure using the corrected Akaike Information Criterion (AICc) to obtain the model with the best trade‐off between parsimony and goodness‐of‐fit criteria. LMM were fitted with maximum‐likelihood for AICc comparison, and final models were refitted using residual maximum‐likelihood for better estimation of variance components (Zuur *et al*., 2009). As abundance data showed high overdispersion, they were modeled using Negative‐Binomial GLMM, after exploring the variance‐to‐mean relationship (Ver Hoef & Boveng, 2007). The value of the dispersion parameter *θ* was estimated using the glmer.nb function in lme4 package (Bates *et al*., 2015).

Usual checks were made to assess model validity (Zuur *et al*., 2010). Global and local Moran's I (package spdep, Bivand & Piras, 2015) were computed from the residuals of every year to make sure that no spatial autocorrelation was left. All points were considered connected, as Cocu *et al*. (2005b) found that spatial autocorrelation was still possible at a distance of 700 km. Spatial weights were computed as the inverse of geographical distance. Absence of temporal autocorrelation was checked by computing an autocorrelation function on the residuals for each trap. To avoid bias due to unbalanced data, each model was refitted on an almost balanced dataset, corresponding to the period between 1989 and 2001 when almost all traps studied were working (Table 1). We checked that *P*‐values and coefficient estimates did not significantly depart from the ones obtained from the model fitted to the full dataset.

We used the method proposed by Nakagawa & Schielzeth (2013) to compute the variance explained by the models (see also Nakagawa & Schielzeth, 2010 for estimation of the variance components). Both marginal variance (variance explained by the fixed effects) and conditional variance (variance explained by both fixed and random effects) were computed. For GLMM,variance is explained on the log scale.

We computed partial regression coefficients of predictor variables, thus allowing comparison of their relative effects despite the potential remaining correlation between them (Bring, 1994). Coefficient absolute values were divided by the sum of absolute values of every coefficient from the model they were taken from, to obtain a value between 0 and 1 (expressed as a percentage). Those standardized coefficients were then summed for each group of variables (climatic, land‐use, and geographical variables and interactions), indicating their relative importance in explaining variation in the dependent variable.

All statistical analyses were carried out using R software 3.3 (R Core Team, 2016) and models were fitted using the lme4 package (Bates *et al*., 2015) and mgcv package (Wood, 2006).

## Results

### Changes in biological and environmental parameters over time

**Figure 2:**
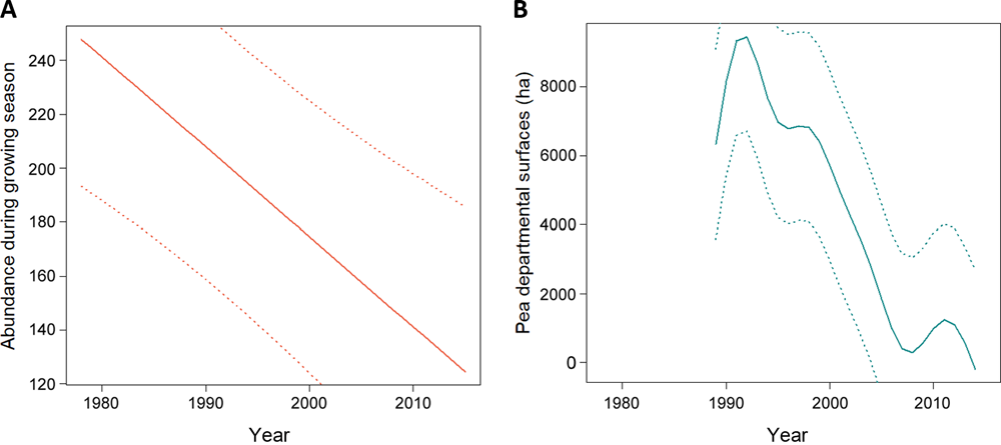
Yearly dynamics, averaged over all traps and species, of: (A) aphid summed abundance during growing season between 1978 and 2015, (B) pea regional area between 1989 and 2015. Curves were obtained using smoothing splines. Dashed lines represent standard errors.

On average, 198 individuals of the five aphid species studied were captured annually in each trap, with high variation across traps (SE = 90) and species (SE = 87). However, average aphid abundance varied following a near linear decreasing trend in time, decreasing by half in 37 years (from 248 ± 54 in 1978 to 124 ± 61 in 2015, see Fig. 2A). In addition, aphid flight initiation (D5C) tended to be recorded earlier every year (notably between 1978 and 1992), advancing on average by 1 month in 37 years (from 165 ± 9 in 1978 to 131 ± 11 in 2015, Fig. 3).

**Figure 3:**
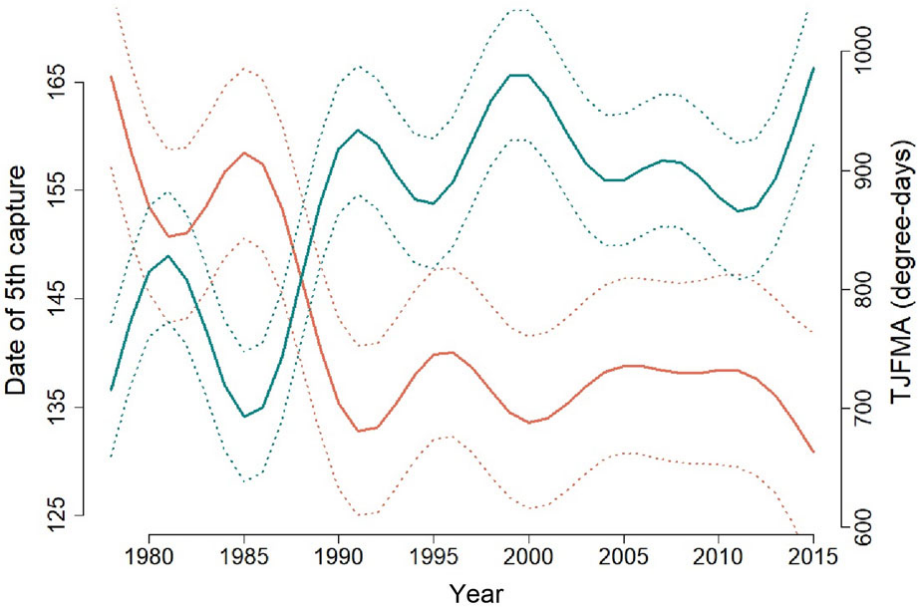
Yearly dynamics of temperature from January to April (in blue) (TJFMA) and aphid date of 5th capture (in red) (D5C), averaged over all species and all traps using smoothing splines. Dashed line represents standard errors.

Regional surfaces covered by protein crops reached a peak in 1991 (9462 ± 2746 ha), then decreased continuously, except for a small peak in 2010 (Fig. 2B). Pea represented the major protein crop, although faba bean area increased in the 2000s (95% pea, 5% faba bean, 0% lupin in 1989; 63% pea, 34% faba bean, and 3% lupin in 2015). On the other hand, temperature variables (TJFMA, TMAY, and TMA16) showed an almost constant increase between 1978 and 2015, except a notable drop between 1983 and 1986. Winter temperature (TJFMA), in particular, showed a remarkable quasi‐symmetric pattern to aphid D5C (Fig. 3). Its average value in degree‐days >0 °C was 716 ± 56 in 1978 and 986 ± 64 in 2015.

**Figure 4:**
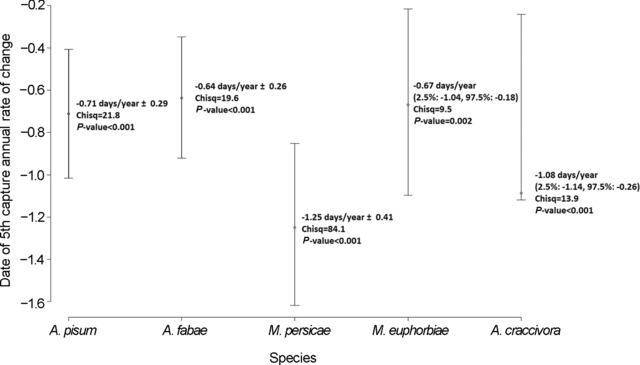
Linear annual rate of advancement for each species date of 5th capture (D5C), corresponding to Linear Mixed Model regression coefficients. Error bars correspond to bootstrapped IC95 (1000 resampling).

Linear mixed models showed a significant increase of TJFMA over time (+6.6 degree‐days/year ± 0.5, *χ* ^2^ = 212.86, *P* < 0 .001) along with a significant advance in aphid D5C (–0.8 day/year ± 0.1, *χ* ^2^ = 84.1, *P* < 0.001). The D5C of all individual species advanced with year, but overlapping coefficients did not allow us to infer significant differences between each species’ annual rate of change (Fig. 4).

### Effects of environmental drivers on aphid abundance and phenology

Many relationships were found between environmental factors (climate, crop area, and geography) and biological variables (abundance and D5C), with different profiles according to species (Tables 3, 4, and S1).

**Table 3:** Crop area, temperature, geographic variables and interactions selected for each abundance model by the stepwise procedure (AICc). The sign (+/–) represents the direction of the relationship between the explanatory variable and the explained variable; ns means the variable was selected but not significant. Fb: faba bean. TJFMA: temperature from January to April in degree‐days >0 °C. TMAY: temperature in May in degree‐days >0 °C. TMA16: temperature in April and May in degree‐days >16 °C. See Table S1 for statistical details

Abundance	All species	*A. pisum*	*A. fabae*	*M. euphorbiae*	*M. persicae*
Pea	+	+	+	+	*ns*
Faba bean		ns			ns
TJFMA	‒	‒	ns	‒	‒
TMAY	‒		ns		
Pea × TJFMA			ns		‒
Fb × TJFMA		ns			+
Pea × TMAY					
Fb × TMAY					
Latitude	‒	‒	‒		
Longitude	+		+	+	ns

**Table 4:** Crop area, temperature, geographic variables, and interactions selected for each D5C model by the stepwise procedure (AICc). The sign (+/–) represents the direction of the relationship between the explanatory variable and the explained variable; ns means the variable was selected but not significant. Fb: faba bean. TJFMA: temperature from January to April in degree‐days >0 °C. TMAY: temperature in May in degree‐days >0 °C. TMA16: temperature in April and May in degree‐days > 16 °C. See Table S1 for statistical details

Date of 5th capture (D5C)	All species	*A. pisum*	*A. fabae*	*M. euphorbiae*	*M. persicae*	*A. craccivora*
Pea	ns	ns	ns	+	ns	+
Faba bean	ns	ns	ns	ns	ns	
TJFMA	‒	‒	‒	‒	‒	‒
TMA16			ns	ns	ns	
Pea × TJFMA			‒	‒		
Fb × TJFMA	ns					
Pea × TMA16	‒		+	+	+	
Fb × TMA16	‒	‒	‒	‒	‒	
Latitude		+			+	+
Longitude	‒	‒	‒	‒	ns	‒

#### Crop area

We found a positive relationship between pea crop area and abundance for aphid species preferring Fabaceae hosts: *A. pisum*, *A. fabae*, and *M. euphorbiae* (Tables 3 and S1). The generalist *M. persicae* showed no response to pea area variation alone. No model could be fitted for *A. craccivora* abundance, due to convergence problems, probably because of lack of data (*N* = 82). Only pea crop area was selected in abundance models and not faba bean area, other than its inclusion in an interaction for *M. persicae* (Tables 3 and S1). Pea area increase was associated with earlier flight initiation (D5C) of *A. craccivora* (–13.1 ± 4.0, *χ* ^2^ = 11.2, *P* < 0.001) and *M. euphorbiae* (−5.3 ± 2.4, *χ* ^2^ = 10.1, *P* < 0.001).

#### Climatic variables

Overall, temperature variables were negatively linked to aphid abundance (i.e., aphids were less abundant when it got warmer) (Tables 3 and S1). Models for each species showed a negative relationship between winter temperature (TJFMA) and aphid abundance *for A. pisum* (−0.22 ± 0.10, *χ* ^2^ = 4.0, *P* < 0.05), *M. euphorbiae* (−0.22 ± 0.10, *χ* ^2^ = 5.2, *P* < 0.05), and *M. persicae* (−0.47 ± 0.12, *χ* ^2^ = 17.2, *P* < 0.001). Temperature in May (TMAY) was retained as a significant variable over all species abundance (–0.17 ± 0.06, *χ* ^2^ = 8.1, *P* < 0.01) but not for a particular species. Every species showed a strong negative response to TJFMA for its D5C (Tables 4 and S1), indicating earlier dates of migration with milder winter temperature. No significant effect of TMA16 (temperature above 16 °C) on D5C was found for any species.

#### Interactions

Interactions between temperature and crop area were related to aphid abundance only for *M. persicae* (Faba bean × TJFMA: 0.27 ± 0.11, *χ* ^2^ = 6.0, *P* < 0.05, Pea × TJFMA: –0.19 ± 0.09, *χ* ^2^ = 4.7, *P* < 0.05). Regarding D5C, such interactions were selected more often, with higher coefficients, than crop area alone in most cases (Tables 3 and S1). The positive interaction Pea × TJFMA was associated with earlier dates of migration for two species (Tables 4 and S1). The interaction between pea area and temperature above 16 °C in March and April (Pea × TMA16) was related to later migration for three species, while the interaction Faba bean × TMA16 was related to earlier migration dates for three species (Tables 4 and S1).

### Geographic variables

Increasing latitude was strongly associated with greater abundance for all aphid species abundance except one (*M. euphorbiae*), and later migration dates of *A. pisum*, *A. craccivora*, and *M. persicae* (Tables 3, 4, and S1). Longitude was positively related to abundance for the “all species” model and was found significant for *A. fabae* (0.47 ± 0.14, *χ* ^2^ = 10.8, *P* < 0.01) and *M. euphorbiae* (0.52 ± 0.14, *χ* ^2^ = 14.3, *P* < 0.001). It also showed a negative relationship with D5C for all species except *M. persicae*. This indicates that aphids are more abundant and appear earlier in the East.

#### Variance decomposition

**Figure 5:**
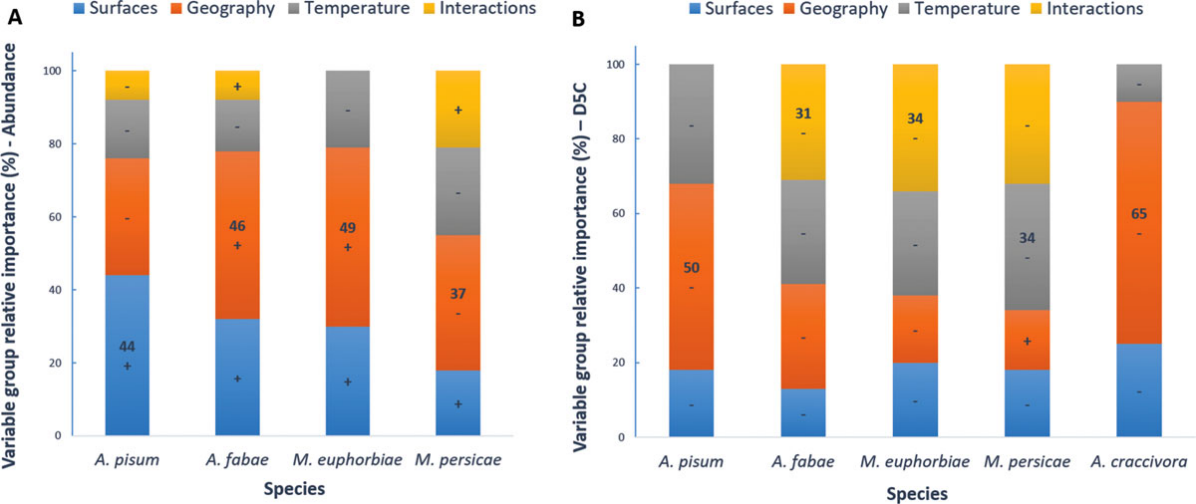
Relative importance of each variable group (crop area, geography, temperature, interactions) in explaining (A) aphid species abundance and (B) date of 5th capture (D5C). “+” or “–” indicate the overall effect of each variable group. They correspond to the sign of the summed coefficients of each variable of this group. Displayed values correspond to the relative importance of the group with the higher %, for each species.

**Abundance**: Climatic, land‐use, and geographic variables explained from 31% to 37% of insect overall and specific abundance (Table S1). For all species, geographic variables better explained abundance compared to other variables, followed by area of Fabaceae protein crop and temperature, except for *M. persicae*, the abundance of which was the least explained by protein crop area (18% relative importance) and *A. pisum* for which they were the most important factors (Fig. 5A). *A. pisum* also had the highest correlation with pea area (0.61 ± 0.10, *χ* ^2^ = 36.7, *P* < 0.001) and the highest ratio of crop area coefficient to other coefficients (44% relative importance). For each aphid species, interactions contributed little to the variance explained by the fixed effects (0–21% relative importance). Overall, crop area had a positive effect on each aphid species’ abundance, while temperature had a negative effect (Fig. 5A). Geographical variables and interactions had variably positive or negative overall effects (Fig. 5A).

**Migration dates (D5C)**: Variance explained by explanatory factors varied from 36% (*M. euphorbiae*) to 73% (*A. pisum*) (Table S1). For all species except *A. craccivora*, TJFMA was the more important variable, although the “temperature group” did not always have the higher relative importance as it contained only one variable. Migration dates were more strongly influenced by geographical factors for *A. pisum* and *A. craccivora*, by interactions between crop area and temperature for *A. fabae* and *M. euphorbiae*, and by temperature alone for *M. persicae* (Fig. 5B). *M. persicae* was the species for which temperature had the highest relative contribution (34% relative importance). Climatic variables had a higher importance than crop area for all species except *A. craccivora* (Fig. 5B). Temperature, crop area, and interaction variable groups all tended to make aphids appear earlier (overall negative effect, Fig. 5B). Relationships between D5C and latitude were negative, and relationships between D5C and longitude were positive (Table 4). As the resulting overall effects of geographical variables were negative, except for *M. persicae* (Fig. 5B), it seems that aphid phenology was more closely related to latitude than longitude.

## Discussion

Both of the biological parameters studied, aphid phenology and abundance, significantly evolved during the last four decades. Aphids advanced their spring migration at a rate varying between 0.6 d/year and 1.2 d/year, while their average abundance diminished by half in 37 years. We showed that climatic and land‐use factors explain a large part of this variation, independently or in interaction. Aphid phenological changes were tightly related to temperature increase, while aphid numbers depended more on regional crop area. Insect responses varied across species however, as legume specialist aphids were more impacted by regional habitat modification than generalists. In addition to these effects, we could show a significant geographical structuring of aphid population characteristics in France.

### Temperature influence on aphid populations

Since 1978, aphid spring migration dates have evolved remarkably symmetrically to winter and early spring temperature. Models indeed showed that temperature at this time of the year was the main factor influencing dates of first flight, at least for four out of five species. This highlights the tight link existing between temperature and aphid phenology, and reinforces previous results that have established a clear relationship between climate change and advancement in aphid migration dates (Harrington *et al*., 1990; Hullé *et al*., 1994; Cocu *et al*., 2005b; Harrington *et al*., 2007; Bell *et al*., 2015). These results indicate that climatic events occurring on winter stages of aphid populations have a major influence in determining spring migration patterns. The five species studied may face two different situations in winter, depending on their reproductive mode, and two distinct mechanisms may explain their response. Aphids may be obligate parthenogens (OP), that is, they overwinter as cold‐sensitive parthenogenetic individuals, or cyclical parthenogens (CP), that is, sexual females lay cold‐resistant eggs that spend winter in diapause (Simon *et al*., 2010). OP population survival and fecundity rates are strongly influenced by temperature, which is often a limiting factor to population growth when it is low (Blackman, 1974). Dates of egg hatch in CP populations may be advanced when temperature increases (Bale *et al*., 2002). Thus, in both cases, warmer winters would lead to greater population size when crops become suitable for aphid colonization. As winged morph formation is positively density‐dependent (Müller *et al*., 2001; Braendle *et al*., 2006), this higher density of early aphid populations on their winter host plants is likely to lead to earlier migrations into crops. In the five species studied here, the proportion of OP and CP lineages is known to change in France through both time and space, depending on climatic factors and winter host availability (Blackman & Eastop, 2000; Frantz *et al*., 2006). Some species are very likely mainly OP (e.g., *A. craccivora*, *M. persicae*), while for others the proportion of OP and CP populations may vary considerably from one year to another, and geographically (notably *A. pisum*, Frantz *et al*., 2006). However, our data did not allow the assigning of each sample to its reproductive mode. The fact that we could not separate OP and CP populations may explain why we did not find significant differences between species’ annual rate of change in flight initiation (even if we found different relative influences of temperature on insect phenology). In future studies, a more precise biogeographical analysis and genetic tools could help consideration of intraspecific variation in life cycles, in order to decipher the relationships between such traits and insect responses to climate change.

Although temperature was important in explaining aphid phenology, its effect on abundance was weaker. However, if climate change seems beneficial for aphid winter populations, it had quite a negative effect on their abundance during the growing season. As we worked on flying individuals only, such a decrease could reflect global reduction in aphid densities or specific reduction in winged morph production. Total population sizes of several aphid species have been shown or predicted to be lowered notably through indirect effects of temperature increase, either bottom‐up (Michaud, 2010) or top‐down (Barton & Ives, 2014; Meisner *et al*., 2014). This may be the case even when direct effects of global change are beneficial for pests (Michaud, 2010), as is expected in France where aphids live most of the time in suboptimal conditions (Hullé *et al*., 2010). Climate change can notably increase parasitism and predation, leading to negative net effects on aphids (Barton & Ives, 2014; Meisner *et al*., 2014), or alter parasitoid–host synchrony (van Nouhuys & Lei, 2004). Natural enemy data and more processed‐based mechanistic models will be needed to test these different assumptions. On the other hand, the negative responses we observed could concern aphid aerial populations only. Several studies indicate that production of winged morphs tends to decrease as temperature gets higher (Müller *et al*., 2001; Braendle *et al*., 2006). Thus, although very useful for studying aphid phenological responses to climate changes, suction trap captures might not be representative enough to study temperature effects on total population abundance. In any case, contrasting responses between aphid phenology and abundance (earlier appearance in crops but lower numbers afterward) underline the importance of within‐season ecological processes influencing winged and wingless aphid population growth rates (Bell *et al*., 2015). Field data on wingless aphids (which are more likely to be predated or parasitized) and natural enemies, as well as a more detailed analysis of aphid seasonal dynamics could help the understanding of such processes.

### Land‐use influence on aphid populations and interactions between land‐use and climate

Protein crop regional area varied considerably between 1989 and 2015. Although it decreased continuously until 2010, it increased slightly in the last studied years, which could be explained by European Union incentive policies during this period (de Visser *et al*., 2014). As expected, we found a positive relationship between protein crop area and aphid species feeding on these crops, indicating that insects become more abundant as their resource density increases. This result confirms an existing relationship between land‐use features and pest numbers at a regional scale (Cocu *et al*., 2005a,b). We found that pea crop area had the greater influence; however other protein crops covered a low area with little variability, and we could have missed some minor effects. The influence of legume crop area on aphid abundance seems to differ among species. Its relative importance was the highest for *Acyrthosiphon pisum*, a legume specialist, and diminished for more generalist species. It was the lowest for *Myzus persicae*, which was the most generalist aphid we studied (Blackman & Eastop, 2000; Hullé *et al*., 2006). This pattern is consistent with the resource concentration hypothesis (Root, 1973), which predicts that herbivore numbers will increase when their resource density increases, but with larger responses from specialized species. This emphasizes the fact that insect traits can be major drivers of their responses to habitat modification, even at a broad scale (Öckinger *et al*., 2010; Andersson *et al*., 2013). Pest differential responses at such a large scale may have deep implications for biotic interactions and lead to important community shifts (Tscharntke *et al*., 2012): here, greater responses of specialists could lead to predominance of these populations in agroecosystems. However, we remain cautious about the influence of the degree of specialization on aphid responses to crop area variation. Indeed, at least two of the species we studied (*A. pisum*, Peccoud *et al*., 2015 and *A. fabae*, Vorburger *et al*., 2017) are actually cryptic complexes of different subspecies or biotypes, each having a specific host range which may not include protein crops. Morphological identification could not allow discrimination of these different subspecies, and we do not know what fraction of our samples corresponded to the ones specialized on pea and other protein crops. Besides, our analysis was carried out on a subset of aphid species, in few locations and with few variables, and further research effort, implying more species and molecular tools to identify subspecies and biotypes, is needed to examine the impact of changes in resource abundance on specialists versus generalists. The apparent contrasting responses of aphid species according to their degree of specialization are however encouraging for future studies willing to explore this relationship further. Trait‐based approaches constitute a promising way to unravel global change impacts on insect interactions and outcomes at the community level (Tylianakis *et al*., 2008; Le Provost *et al*., 2017).

In contrast with aphid abundance, crop area was less important than temperature in explaining migration dates, although we found some minor effects. However, significant interactions indicate that combined effects of climate and land‐use may occur. Over all species, it seems that a positive interaction between temperature in winter and crop area leads to earlier aphid flight initiation, meaning that effects of temperature would become greater as crop area increases. Lantschner *et al*. (2014) suggested that growth rate acceleration of a pine pest in discrete locations, associated to climate change, could increase even more when its host area expands, because it allows a quick proliferation of this species in new forests. The same synergistic effect can be envisaged for aphids. Temperature increase would allow earlier production of winged morphs, whose probability of finding host‐plants would become greater as crop area increases. Aphid populations on crops would then increase and produce new winged morphs faster, thus quickening aphid proliferation at a regional scale (thus increasing the probability of catching them earlier). However, we remain cautious about this interpretation as we also found that interaction effects on particular species led to later migration dates. Interactions between climate and land‐use factors can have multiple facets and are difficult to interpret without very large amounts of data; nonetheless it is important to consider them to partition variation due to land‐use and climate effects in a precise way (Oliver & Morecroft, 2014). Our results underline the importance of considering such possible interactions in order to have a better understanding of intricate effects acting on insect dynamics in further studies.

### Geography influence on aphid populations

Cocu *et al*. (2005b) showed that geographical variation of climate, land‐use, and aphid abundance may broadly occur at the same regional scale. Our results tend to confirm that such variables explain a substantial part of the spatial structure of aphid abundance, as well as phenology. However, despite sizeable effects of temperature and crop area, geographical variables still had the highest relative importance in explaining the abundance of three out of five species and the dates of first flight of two out of five species. Latitude and longitude effects indicate that, the other variables being held constant, insects are more abundant and appear earlier in the South and in the East of France. Model coefficient values notably show a strong latitudinal effect on aphid population parameters. Geographical factors may cover many other spatially structured processes that act on aphid abundance and phenology. For simplicity purposes, we restricted ourselves to three temperature variables, but many other climatic factors can affect insect dynamics, such as precipitation patterns or temperature seasonality (Newman, 2005; Diehl *et al*., 2013). For instance, France has an important north–south climatic gradient, with a singular Mediterranean climate in the south, which cannot be totally captured in only a few parameters. In addition, suction trap captures are highly correlated with farming practices at different scales (Benton *et al*., 2002), which may differ between regions. Agricultural intensity or insecticide treatments could be major factors of interest (Tscharntke *et al*., 2005). Finally, we considered land‐use factors through raw crop area data, but had no information about landscape structure or functionality, which can mask important geographical features influencing pest abundance (Veres *et al*., 2013).

### Aphids facing global changes: future directions

In the last four decades, aphids in France have advanced their spring migration by one month. This trend also holds true for the United Kingdom (Bell *et al*., 2015) and is likely to be worldwide, and to continue with global warming. Our work showed that land‐use drivers also explain a significant part of large‐scale aphid population patterns. In a context of increasing protein crop production (de Visser *et al*., 2014), growing pest populations are to be expected, possibly favoring specialists. More generally, the importance of land‐use parameters, interaction between environmental factors, and the large part of aphid variation that remains unexplained underline the need to consider a larger panel of global change drivers than climate alone, in order to have an integrative view of insect population responses. Besides, cascading consequences of insect pest responses to global changes on community structure and ecosystem functioning constitute a promising area of research, and major regional patterns still need to be identified.

## Disclosure

The authors declare that there is no conflict of interest regarding this study or the publication of this article.

## Supporting information


**Table S1**. Variables selected for each model, with associated coefficients ± SE, chi‐squared statistics (Wald tests) and *P* values. *P < 0.05, **P < 0.01, ***P < 0.001. In bold: significant variables. (A) Abundance as a response variable. (B) Date of 5th capture as a response variable. Third column indicates variance explained by the models. Marginal variance: variance explained by the fixed effects. Conditional variance: variance explained by fixed and random effects (Nakagawa & Schielzeth, 2013). Fourth column indicates the number of trap‐years used to fit the models. TJFMA: Temperature from January to April in degree‐days >0 °C. TMAY: temperature in May in degree‐days >0 °C. TMA16: temperature in April and May in degree‐days >16 °C.
