# Supplementary material for: Relative importance of long‐term changes in climate and land‐use on the phenology and abundance of legume crop specialist and generalist aphids
Source: Insect Sci. 2018 May 17;26(5):881–96. doi: 10.1111/1744-7917.12585 (PMC7379299; doi:10.1111/1744-7917.12585)
Supplement: Supplementary file 1 — Table S1. Variables selected for each model, with associated coefficients ± SE, chi‐squared statistics (Wald tests) and P values. *P < 0.05, **P < 0.01, ***P < 0.001. In bold: significant variables. (A) Abundance as a response variable. (B) Date of 5th capture as a response variable. Third column indicates variance explained by the models. Marginal variance: variance explained by the fixed effects. Conditional variance: variance explained by fixed and random effects (Nakagawa & Schielzeth, 2013). Fourth column indicates the number of trap‐years used to fit the models. TJFMA: Temperature from January to April in degree‐days >0 °C. TMAY: temperature in May in degree‐days >0 °C. TMA16: temperature in April and May in degree‐days >16 °C. [file INS-26-881-s001.doc]

## Supplementary materials

**Table S1** Variables selected for each model, with associated coefficients *± SE*, Chi-squared statistics (Wald tests) and p-values. * P-value < 0.05, ** P-value < 0.01, *** P-value < 0.001. In bold: significant variables. **A.** Abundance as a response variable. **B.** Date of 5^th^ capture as a response variable. Third column indicates variance explained by the models. Marginal variance: variance explained by the fixed effects. Conditional variance: variance explained by fixed and random effects (Nakagawa & Schielzeth 2013). Fourth column indicates the number of trap-years used to fit the models. TJFMA: Temperature from January to April in degree-days >0^o^C. TMAY: Temperature in May in degree-days >0^o^C. TMA16: Temperature in April and May in degree-days >16^o^C.

| **A. Abundance** | **Present variables in the selected model**  (coefficient estimate ± SE, Chisq) | **Variance explained** | | **Number of**  **trap-years** |
| --- | --- | --- | --- | --- |
|  |  | **marginal** | **conditional** |  |
| All species | **Pea** (0.39 ± 0.06, Chisq=38.3) ***  **TJFMA** (-0.29 ± 0.07, Chisq=18.0) ***  **TMAY** (-0.17 ± 0.06, Chisq=8.1) **  **Latitude** (-0.38 ± 0.07, Chisq=32.5) ***  **Longitude** (0.42 ± 0.08, Chisq=28.3) *** | 36% | 65% | 640 |
| *Acyrthosiphon pisum* | **Pea** (0.61 ± 0.10, Chisq=36.7) ***  **TJFMA** (-0.22 ± 0.10, Chisq=4.0) *  **Latitude** (-0.44 ± 0.18, Chisq=16.5) ***  Faba bean (0.002 ± 0.09, Chisq=2.7, *P* = 0.10)  Faba bean x TJFMA (-0.12 ± 0.07, Chisq=2.4, *P* = 0.12) | 31% | 51% | 132 |
| *Aphis fabae* | **Pea** (0.51 ± 0.12, Chisq=14.2) ***  **Latitude** (-0.27 ± 0.12, Chisq=5.3) *  **Longitude** (0.47 ± 0.14, Chisq=10.8) **  TJFMA (-0.07 ± 0.09, Chisq=1.4, *P* = 0.23)  TMAY (-0.16 ± 0.09, Chisq=3.36, *P* = 0.07)  Pea x TJFMA (0.12 ± 0.06, Chisq=3.8, *P* = 0.052) | 37% | 48% | 124 |
| *Macrosiphum euphorbiae* | **Pea** (0.32 ± 0.10, Chisq=9.4) **  **TJFMA** (-0.22 ± 0.10, Chisq=5.2) *  **Longitude** (0.52 ± 0.14, Chisq=14.3) *** | 31% | 58% | 133 |
| *Myzus persicae* | **TJFMA** (-0.47 ± 0.12, Chisq=17.2) ***  **Faba bean x TJFMA** (0.27 ± 0.11, Chisq=6.0) *  **Pea x TJFMA** (-0.19 ± 0.09, Chisq=4.7) *  **Latitude** (-0.58 ± 0.12, Chisq=26.0) ***  Pea (-0.03 ± 0.12, Chisq=1.0, *P* = 0.32)  Faba bean (0.41 ± 0.13, Chisq=3.6, *P* = 0.06)  Longitude (0.24 ± 0.14, Chisq=3.0, *P* = 0.09) | 37% | 48% | 127 |

| **B. Date of 5th capture** | **Present variables in the selected model**  (coefficient estimate ± SE, Chisq) | **Variance explained** | | **Number of trap-years** |
| --- | --- | --- | --- | --- |
|  |  | **marginal** | **conditional** |  |
| All species | **TJFMA** (-9.9 ± 1.1, Chisq=81.8) ***  **Faba bean x TMA16** (-3.5 ± 1.5, Chisq=7.5) **  **Pea x TMA16** (-3.5 ± 1.5, Chisq=5.3) *  **Longitude** (-8.4 ± 2.5, Chisq=11.4) ***  Faba bean (-4.0 ± 1.5, Chisq=0.1, *P* = 0.73)  Pea (-1.1 ± 1.6, Chisq=0.0007, *P* = 0.97)  Faba bean x TJFMA (-1.3 ± 0.9, Chisq=2.3, *P* = 0.13) | 41% | 71% | 515 |
| *Acyrthosiphon pisum* | **TJFMA** (-8.69 ± 1.4, Chisq=36.7) ***  **Latitude** (6.5 ± 2.1, Chisq=9.9) **  **Longitude** (-7.0 ± 2.0, Chisq=11.8) ***  Pea (-2.9 ± 1.3, Chisq=2.2, *P* = 0.10)  Faba bean (-1.9 ± 1.8, Chisq=2.8, *P* = 0.13) | 73% | 83% | 131 |
| *Aphis craccivora* | **Pea** (-13.1 ± 4.0, Chisq=11.2) ***  **TJFMA** (-5.0 ± 2.4, Chisq=4.4) *  **Latitude** (11.1 ± 4.5, Chisq=6.0) *  **Longitude** (-19.3 ± 5.1, Chisq=14.6) *** | 69% | 88% | 51 |
| *Aphis fabae* | **TJFMA** (-8.3 ± 1.4, Chisq=29.6) ***  **Faba bean x TMA16** (-4.0 ± 1.8, Chisq=5/0) *  **Pea x TJFMA** (-2.5 ± 1.1, Chisq=5.2) *  **Pea x TMA16** (3.6 ± 1.2, Chisq=9.4) **  **Longitude** (-9.0 ± 2.4, Chisq=14.0) ***  Pea (-2.1 ± 2.2, Chisq=2.4, *P* = 0.12)  Faba bean (-2.2 ± 2.0, Chisq=0.9, *P* = 0.34)  TMA16 (-1.0 ± 1.3, Chisq=0.77, *P* = 0.38) | 60% | 78% | 117 |
| *Macrosiphum euphorbiae* | **Pea** (-5.3 ± 2.4, Chisq=10.1) **  **TJFMA** (-10.0 ± 1.9, Chisq=22.0) ***  **Faba bean x TMA16** (-6.6 ± 2.2, Chisq=9.4) **  **Pea x TJFMA** (-3.4 ± 1.4, Chisq=6.1) *  **Pea x TMA16** (4.1 ± 1.4, Chisq=8.3) **  **Longitude** (-7.3 ± 2.0, Chisq=14.0) ***  Faba bean (-2.8 ± 2.0, Chisq=1.1, *P* = 0.29)  TMA16 (2.1 ± 1.8, Chisq=1.8, *P* = 0.18) | 36% | 62% | 95 |
| *Myzus persicae* | **TJFMA** (-14.4 ± 1.77, Chisq=63.5) ***  **Faba bean x TMA16** (-4.9 ± 2.2, Chisq=5.1, *P* = 0.12) *  **Pea x TMA16** (3.1 ± 1.2, Chisq=6.3, *P* = 0.28) *  **Latitude** (4.3 ± 1.8, Chisq=5.7) *  Faba bean (-9.3 ± 2.7, Chisq=2.2, *P* = 0.14)  Pea (1.7 ± 2.2, Chisq=1.2, *P* = 0.26)  TMA16 (-0.2 ± 1.7, Chisq=0.02, *P* = 0.88)  Longitude (-2.8 ± 1.8, Chisq=2.5, *P* = 0.11) | 72% | 78% | 126 |
